# Supplementary material for: Pyroptotic cell corpses are crowned with F-actin-rich filopodia that engage CLEC9A signaling in incoming dendritic cells
Source: Nat Immunol. 2024 Dec 4;26(1):42–52. doi: 10.1038/s41590-024-02024-3 (PMC11695261; doi:10.1038/s41590-024-02024-3)
Supplement: Supplementary file 1 — Reporting Summary [file 41590_2024_2024_MOESM1_ESM.pdf]

nature portfolio

Corresponding author(s): *Double-anonymous peer review submissions: write DAPR and your manuscript number here instead of author names.*

Last updated by author(s): 02/09/2024

Reporting Summary

Nature Portfolio wishes to improve the reproducibility of the work that we publish. This form provides structure for consistency and transparency in reporting. For further information on Nature Portfolio policies, see our Editorial Policies and the Editorial Policy Checklist.

Statistics

For all statistical analyses, confirm that the following items are present in the figure legend, table legend, main text, or Methods section.

n/a	Confirmed	
☐	☒	The exact sample size (*n*) for each experimental group/condition, given as a discrete number and unit of measurement
☒	☐	A statement on whether measurements were taken from distinct samples or whether the same sample was measured repeatedly
☐	☒	The statistical test(s) used AND whether they are one- or two-sided<br>*Only common tests should be described solely by name; describe more complex techniques in the Methods section.*
☒	☐	A description of all covariates tested
☐	☒	A description of any assumptions or corrections, such as tests of normality and adjustment for multiple comparisons
☐	☒	A full description of the statistical parameters including central tendency (e.g. means) or other basic estimates (e.g. regression coefficient) AND variation (e.g. standard deviation) or associated estimates of uncertainty (e.g. confidence intervals)
☐	☒	For null hypothesis testing, the test statistic (e.g. *F*, *t*, *r*) with confidence intervals, effect sizes, degrees of freedom and *P* value noted<br>*Give P values as exact values whenever suitable.*
☒	☐	For Bayesian analysis, information on the choice of priors and Markov chain Monte Carlo settings
☒	☐	For hierarchical and complex designs, identification of the appropriate level for tests and full reporting of outcomes
☒	☐	Estimates of effect sizes (e.g. Cohen's *d*, Pearson's *r*), indicating how they were calculated

Our web collection on statistics for biologists contains articles on many of the points above.

Software and code

Policy information about availability of computer code

Data collection	Immunofluorescence: Zeiss Zen 2012 software v2.3<br>Plate reader Tecan: i-controlTM (v2.0)<br>LLAMA visualisation software: Lefevre, J.G. et al. LLAMA: a robust and scalable machine learning pipeline for analysis of large scale 4D microscopy data: analysis of cell ruffles and filopodia. BMC Bioinformatics 22, 410 (2021).<br>Flow cytometry: BD FACSDiva (v9.0)
Data analysis	Prism (v9/10)<br>Arivis Vision4D (v4.1.1)<br>Fiji/ImageJ (v1.52k-1.54f)<br>Microvolution Deconvolution: https://www.microvolution.com/<br>FlowJo (v.10.9)

For manuscripts utilizing custom algorithms or software that are central to the research but not yet described in published literature, software must be made available to editors and reviewers. We strongly encourage code deposition in a community repository (e.g. GitHub). See the Nature Portfolio guidelines for submitting code & software for further information.

Data

Policy information about availability of data

All manuscripts must include a data availability statement. This statement should provide the following information, where applicable:

- Accession codes, unique identifiers, or web links for publicly available datasets
- A description of any restrictions on data availability
- For clinical datasets or third party data, please ensure that the statement adheres to our policy

The data that support the findings of this study are available from the corresponding author

Research involving human participants, their data, or biological material

Policy information about studies with human participants or human data. See also policy information about sex, gender (identity/presentation), and sexual orientation and race, ethnicity and racism.

Reporting on sex and gender	Human monocyte-derived macrophages (HMDM) were produced from buffy coats from blood donations to the Australian Red Cross Blood Service from anonymous, informed and consenting adults. As a consequence, sex characteristics of the donor material were not known.
Reporting on race, ethnicity, or other socially relevant groupings	Race, ethnicity, and other characateristics of the blood donors were not provided in accordance with the ethical approval.
Population characteristics	See above.
Recruitment	Anonymous volunteers
Ethics oversight	Ethical approval for all experiments involving human blood products was approved by the Research Ethics and Integrity Committee at the University of Queensland (2024/HE000645 and 2016/HE0001044)

Note that full information on the approval of the study protocol must also be provided in the manuscript.

Field-specific reporting

Please select the one below that is the best fit for your research. If you are not sure, read the appropriate sections before making your selection.

☒ Life sciences ☐ Behavioural & social sciences ☐ Ecological, evolutionary & environmental sciences

For a reference copy of the document with all sections, see nature.com/documents/nr-reporting-summary-flat.pdf

Life sciences study design

All studies must disclose on these points even when the disclosure is negative.

Sample size	No statistical method was used to determine sample size. Sample size for image quantification experiments was based on technical feasibility and previously published studies (e.g. PMID: 29432122, PMID: 30150290).
Data exclusions	No data points were excluded from analyses.
Replication	Replications were successful. Where possible, counter assays (e.g. accompanying cell death assays) were used to confirm consistency of responses in cells used for imaging.<br>In vitro experiments; each experiments was performed in n=3 technical replicate, mean of each biological experiments were pooled, and each experiment was performed with at least n=3 biological replicates.<br>In vivo experiments; cohort of 3-4 mice were used for each individual experiments, with n=3 biological replicates
Randomization	For imaging experiments, regions of interest were randomly chosen for quantification. Otherwise, randomisation was not applicable as cells from the same source or differentiation were split into different conditions and/or stimulations.
Blinding	The number of projections per cell was quantified by manual counting by a blinded operator.<br>For in vivo experiments; mice genotypes were unidentified and randomly allocated to groups. Imaging of peritoneal lavage cells were performed from another researcher on unidentified samples.<br>For cell death analysis (LDH release) and flow cytometry analysis (p-Syk, and DC activation) no blinding was performed

Reporting for specific materials, systems and methods

We require information from authors about some types of materials, experimental systems and methods used in many studies. Here, indicate whether each material, system or method listed is relevant to your study. If you are not sure if a list item applies to your research, read the appropriate section before selecting a response.

Materials & experimental systems

n/a	Involved in the study
☐	☒ Antibodies
☐	☒ Eukaryotic cell lines
☒	☐ Palaeontology and archaeology
☐	☒ Animals and other organisms
☒	☐ Clinical data
☒	☐ Dual use research of concern
☒	☐ Plants

Methods

n/a	Involved in the study
☒	☐ ChIP-seq
☐	☒ Flow cytometry
☒	☐ MRI-based neuroimaging

Antibodies

Antibodies used

anti-ASC (1:200, N15; Santa-Cruz; 1:800, 67824S, Cell Signaling Technology), anti-MYO10 (1:100, AB224120, Abcam), anti-phospo-SYK (1:100, 2711S, Cell Signaling Technology), anti-Casp3p17 (1:200, 9661, Cell Signaling Technology), and anti-V5 (1:200, AB27671, Abcam). PE anti-mouse p-Syk (1:50, clone I120-722, BD Biosciences #558529), BV711 anti-mouse CD24 (1:800, clone M1/69, BD Biosciences #563450), APC anti-mouse XCR1 (1:200, clone ZET, Biolegend #148206), and BUV395 anti-mouse CD80 (1:200, clone 16-10A1, BD Biosciences #740246), FITC anti-mouse CD11c (1:200, clone N418, Biolegend #117306), PerCP-eFluor 710 anti-mouse SIRP alpha (1:400, clone P84, Invitrogen #46-1721-82), PE/Cyanine7 anti-mouse CD24 (1:400, clone M1/69, Biolegend #101822), PE anti-mouse CD86 (1:400, clone GL-1, Biolegend #105008), BV711 anti-mouse CD80 (1:200, clone 16-10A1, Biolegend #104743), APC anti-mouse I-A/I-E (MHCII) (1:200, clone M5/114.15.2, Biolegend #107614), and Live/Dead Fixable Violet Dead, (1:400, ThermoFisher # L34964), DAPI (0.1µg/mL, Sigma Aldrich #D9542), Phalloidin-iFluor 405 (1:40, Abcam #AB176752), Alexa Fluor 488 Phalloidin (1:40, Invitrogen #A12379), Alexa Fluor 647 Phalloidin (1:40, Invitrogen #A22287), anti-rabbit Alexa Fluor 594 (1:500, Invitrogen #A32740), donkey anti-mouse Alexa Fluor 488 (1:500, Invitrogen #A21202), and goat anti-rabbit Aexa Fluor 647 (1:500, Invitrogen #A32733).

Validation

anti-ASC (Santa Cruz): https://www.scbt.com/de/p/asc-antibody-n-15 - this antibody has been discontinued

anti-ASC (Cell Signaling Technology): https://www.cellsignal.com/products/primary-antibodies/asc-tms1-d2w8u-rabbit-mab/67824, antibody has been validate by Cell Signaling Technology for mouse cell lines J774.A and Raw264.7 cells for Western blot and Flow cytometry. Paraffin-embedded mouse brain, mouse thymus or mouse colon for immunohistochemistry and mouse Tg2576 brain, mouse primary bone marrow-derived macrophage for immunofluorescent

anti-MYO10: https://www.abcam.com/en-us/products/primary-antibodies/myo10-antibody-ab224120#application=icc-if
antibody has been validated by Abcam on human cell line HeLa for immunofluorescence

anti-phospho-Syk (optimised for IF and FC, using positive and negative controls for SYK phosphorylation): https://www.cellsignal.com/products/primary-antibodies/phospho-syk-tyr525-526-antibody/2711 and has been validated by Cell Signaling Technology on human Ramos cells for Western Blot.

anti-Casp3p17: https://www.cellsignal.com/products/primary-antibodies/cleaved-caspase-3-asp175-antibody/9661
antibody has been validated by Cell Signaling Technology on human and mouse cell lines HeLA, NiH/3T3, C6 cells, Jurkat cells for Western Blot. Paraffin-embedded human tonsils, mouse embryos were used for validation for immunohistochemistry. HT-29 cells were used for validation of immunofluorescence.

anti-V5: https://www.abcam.com/en-us/products/primary-antibodies/v5-tag-antibody-sv5-pk1-ab27671
antibody has been validated by Abcam on transfected cell lysates (simian virus 5 strain W3) for Western blot.

PE anti-mouse p-Syk (clone I120-722, BD Biosciences #558529), https://www.bdbiosciences.com/en-au/products/reagents/flow-cytometry-reagents/research-reagents/single-color-antibodies-ruo/pe-mouse-anti-syk-py348.558529
antibody has been validated by BD Bioscience on human peripheral blood lymphocytes for flow cytometry, mouse testing is in development by BD Bioscience

BV711 anti-mouse CD24 (clone M1/69, BD Biosciences #563450), https://www.bdbiosciences.com/en-au/products/reagents/flow-cytometry-reagents/research-reagents/single-color-antibodies-ruo/bv711-rat-anti-mouse-cd24.563450
antibody has been validated by BD Bioscience on mouse spleenocytes for flow cytometry

APC anti-mouse XCR1 (clone ZET, Biolegend #148206), https://www.biolegend.com/fr-ch/products/apc-anti-mouse-rat-xcr1-antibody-10222
antibody has been validated by Biolegend on mouse spleenocytes for flow cytometry

BUV395 anti-mouse CD80 (clone 16-10A1, BD Biosciences #740246), https://www.bdbiosciences.com/en-au/products/reagents/flow-cytometry-reagents/research-reagents/single-color-antibodies-ruo/buv395-hamster-anti-mouse-cd80.740246
antibody has been validated by BD Bioscience on mouse transfected cell lines

FITC anti-mouse CD11c (clone N418, Biolegend #117306), https://www.biolegend.com/fr-ch/products/fitc-anti-mouse-cd11c-antibody-1815
antibody has been validated by Biolegend on mouse spleenocytes for flow cytometry

PerCP-eFluor 710 anti-mouse SIRP alpha (clone P84, Invitrogen #46-1721-82), https://www.thermofisher.com/antibody/product/CD172a-SIRP-alpha-Antibody-clone-P84-Monoclonal/46-1721-82
antibody has been validated by ThermoFisher on mouse bone marrow cells and bovine PBMC for flow cytometry

PE/Cyanine7 anti-mouse CD24 (clone M1/69, Biolegend #101822), https://www.biolegend.com/fr-ch/products/pe-cyanine7-anti-mouse-cd24-antibody-3862
antibody has been validated by Biolegend on mouse spleenocytes for flow cytometry

PE anti-mouse CD86 (clone GL-1, Biolegend #105008), https://www.biolegend.com/fr-ch/products/pe-anti-mouse-cd86-antibody-256
antibody has been validated by Biolegend on mouse spleenocytes for flow cytometry

BV711 anti-mouse CD80 (clone 16-10A1, Biolegend #104743), https://www.biolegend.com/fr-ch/products/brilliant-violet-711-anti-mouse-cd80-antibody-17823
antibody has been validated by Biolegend on mouse spleenocytes for flow cytometry

APC anti-mouse I-A/I-E (MHCII) (clone M5/114.15.2, Biolegend #107614), https://www.biolegend.com/fr-ch/products/apc-anti-mouse-i-a-i-e-antibody-2488
antibody has been validated by Biolegend on mouse spleenocytes for flow cytometry

Live/Dead Fixable Violet Dead, (ThermoFisher # L34964), https://www.thermofisher.com/order/catalog/product/L34964
antibody has been validated by ThermoFisher on Jurkat cells for flow cytometry

Phalloidin-iFluor 405 (Abcam #AB176752), https://www.abcam.com/en-us/products/reagents/phalloidin-ifluor-405-reagent-ab176752
antibody has been validated by Abcam on bovine BFA cell lines for immunofluorescence

Alexa Fluor 488 Phalloidin (Invitrogen #A12379), https://www.thermofisher.com/order/catalog/product/A12379
antibody has been validated by ThermoFisher on human dermal fibroblasts, bovine pulmonary artery endothelial cells, HeLA cells line for immunofluorescence

Alexa Fluor 647 Phalloidin (Invitrogen #A22287), https://www.thermofisher.com/order/catalog/product/A22287
antibody has been validated by ThermoFisher on multiple cells lines including human A549, BPAE, HCASM, HeLA, U2OS and HUVECs for immunofluorescence

goat anti-rabbit Alexa Fluor 594 (Invitrogen #A32740), https://www.thermofisher.com/antibody/product/Goat-anti-Rabbit-IgG-H-L-Highly-Cross-Adsorbed-Secondary-Antibody-Polyclonal/A32740
antibody has been validated by ThermoFisher using positive MCF10A and negative A-431 cell models as well as different cell lines for immunofluorescence

donkey anti-mouse Alexa Fluor 488 (Invitrogen #A21202), https://www.thermofisher.com/antibody/product/Donkey-anti-Mouse-IgG-H-L-Highly-Cross-Adsorbed-Secondary-Antibody-Polyclonal/A-21202
antibody has been validated by ThermoFisher using different cell lines (MDCK), and primary cells including rat primary cortical neutrons or zebrafsih for immunofluorescence

goat anti-rabbit Aexa Fluor 647 (Invitrogen #A32733) https://www.thermofisher.com/antibody/product/Goat-anti-Rabbit-IgG-H-L-Highly-Cross-Adsorbed-Secondary-Antibody-Polyclonal/A32733.
antibody has been validated by ThermoFisher using different cell lines (A549, THP-1, HeLa, MCF7, HEK-293), and primary cells including primary cortical neutrons or rat brain sections for immunofluorescence

Eukaryotic cell lines

Policy information about cell lines and Sex and Gender in Research

Cell line source(s)	RAW 264.7 (Mouse, male): American Type Culture Collection TIB-71, https://www.atcc.org/products/tib-71<br>HBEC (human): https://www.atcc.org/products/pcs-300-010<br><br>Platinum-E (Plat-E) cell line (human): HMorita, S., Kojima, T. & Kitamura, T. Plat-E: an efficient and stable system for transient packaging of retroviruses. Gene Ther 7, 1063–1066 (2000). https://doi.org/10.1038/sj.gt.3301206, kindly provided by Prof. Matt Sweet, IMB, The University of Queensland, Brisbane, Australial<br><br>HEK293T (human, female): https://www.cellosaurus.org/CVCL_0063
Authentication	Cell lines were not authenticated.
Mycoplasma contamination	All cell lines used were Mycoplasma negative Cells were routinely tested for Mycoplasma contamination via Mycoalert kits or PCR.
Commonly misidentified lines<br>(See ICLAC register)	No commonly misidentified cell lines were used in this study.

Animals and other research organisms

Policy information about studies involving animals; ARRIVE guidelines recommended for reporting animal research, and Sex and Gender in Research

Laboratory animals	Wildtype (C57BL/6J, or littermate controls as appropriate), Casp1C284A/C284A (B6J-Casp1C284Aem1Ksc CR; generated in-house), Casp11-/- (B6.Casp4tm; backcrossed to C57BL/6J), Gsdmd-/- (C57BL/6N-Gasdmdem1Vmd; backcrossed to C57BL/6J), Ninj1-/- (C57BL/6N-Ninj1-ENU-KO) and Clec9a-GFP knock-in (Jackson, B6(Cg)-Clec9atm1.1Crs/J) mice were used in this study. Mice were bred in-house and housed in specific pathogen-free conditions at the University of Queensland. 6-20 week-old male and female animals were sex- and aged-matched for each experiment. Mice were kept at 12 hour dark/light cycle at ambient temperature with ad libitum feeding and water.
Wild animals	The study did not involve wild animals.
Reporting on sex	Findings apply to both sexes. Bone marrow for derivation of primary macrophages was harvested from both male and female mice. LPS challenge in vivo was performed only one female mice due to co-housing of different genotypes.
Field-collected samples	The study did not involve samples collected from the field.
Ethics oversight	All experiments involving mice were approved by The University of Queensland Molecular Biosciences Animal Ethics Committee (2021/AE000419, 2023/AE000019, 2023/AE000020).

Note that full information on the approval of the study protocol must also be provided in the manuscript.

Plants

Seed stocks	*Report on the source of all seed stocks or other plant material used. If applicable, state the seed stock centre and catalogue number. If plant specimens were collected from the field, describe the collection location, date and sampling procedures.*
Novel plant genotypes	*Describe the methods by which all novel plant genotypes were produced. This includes those generated by transgenic approaches, gene editing, chemical/radiation-based mutagenesis and hybridization. For transgenic lines, describe the transformation method, the number of independent lines analyzed and the generation upon which experiments were performed. For gene-edited lines, describe the editor used, the endogenous sequence targeted for editing, the targeting guide RNA sequence (if applicable) and how the editor was applied.*
Authentication	*Describe any authentication procedures for each seed stock used or novel genotype generated. Describe any experiments used to assess the effect of a mutation and, where applicable, how potential secondary effects (e.g. second site T-DNA insertions, mosiacism, off-target gene editing) were examined.*

Flow Cytometry

Plots

Confirm that:

- [ ] The axis labels state the marker and fluorochrome used (e.g. CD4-FITC).
- [x] The axis scales are clearly visible. Include numbers along axes only for bottom left plot of group (a 'group' is an analysis of identical markers).
- [x] All plots are contour plots with outliers or pseudocolor plots.
- [x] A numerical value for number of cells or percentage (with statistics) is provided.

Methodology

Sample preparation	For analysing p-Syk signalling in cDC1 dendritic cells; Flt3L-BMDCs were washed with PBS and immediately fixed with 1% PFA for 20 minutes at room temperature. Cells were spin down at 500 g for 5 minutes and resuspended in pre-chilled 300 µL True-Phos Perm Buffer (425401, Biolegend) and incubated at -20C for 45 minutes. Cells were washed with PBS + 1% FBS and spun at 2000 g for 5 minutes. Flt3L-BMDCs were stained for 45 minutes at 4°C in PBS with 1% FBS containing the following fluorescently conjugated antibodies and dyes.<br>For analysing cDC1 dendritic cell activation, Flt3L-BMDCs were washed and stained for 20 minutes at 4°C in MACS buffer (PBS with 1% FBS, 2 mM EDTA) containing the following fluorescently conjugated antibodies and dye.
Instrument	BD LSRFortessa X-20 (BD Biosciences) and FACSymphony A5 SE (BD Biosciences)
Software	Data acquisition: BD FACSDiva v9.0<br>Data analysis: FlowJo v.10.9
Cell population abundance	Total of 20'000 cells in SSC/FSC were acquired
Gating strategy	For analysing p-Syk signalling in cDC1 dendritic cells; Total cells SSC-A/FSC-A -> Single cells FSC-H/FSC-A -> XCR1+ CD24/XCR1

Gating strategy

-> p-Syk pos Histogram Count/p-Syk
For analysing cDC1 dendritic cell activation; Total cells SSC-A/FSC-A -> Single cells FSC-H/FSC-A -> Live cells FSC-H/Live-Dead -> Dendritic cells CD11c/CD24 -> cDC1 CD24/SIRPa -> Activation CD24/CD80 or CD24/CD86 or CD24/MHCII

☒ Tick this box to confirm that a figure exemplifying the gating strategy is provided in the Supplementary Information.
