# Supplementary figures and images for: Pyroptotic cell corpses are crowned with F-actin-rich filopodia that engage CLEC9A signaling in incoming dendritic cells
Source: Nat Immunol. 2024 Dec 4;26(1):42–52. doi: 10.1038/s41590-024-02024-3 (PMC11695261; doi:10.1038/s41590-024-02024-3)

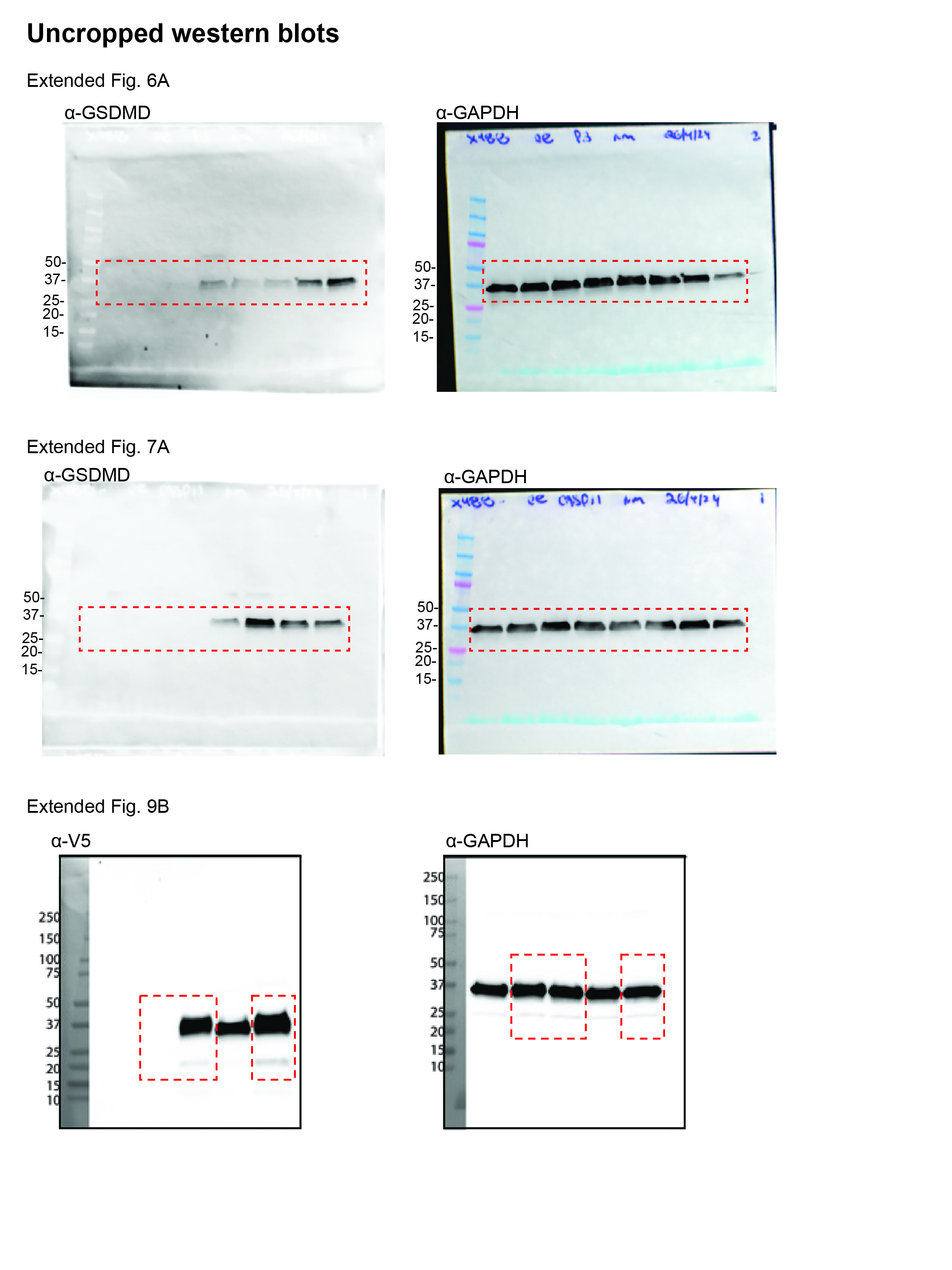

Supplement: Supplementary file 13 — Unprocessed western blots for Extended Data Figs. 6, 7 and 9. [file 41590_2024_2024_MOESM13_ESM.jpg]
